# Environmental Exposures and Oxidative Stress in Retinal and Optic Nerve Diseases: Mechanisms, Consequences, and Therapeutic Opportunities

**DOI:** 10.3390/antiox15030281

**Published:** 2026-02-25

**Authors:** Jacob K. Roberson, Anais N. Bauer, Anahy Lopez-Ramirez, Daniel B. Jenness, Sebastian Cruz Zayas, Jessica N. Cooke Bailey, Tracey L. Woodlief

**Affiliations:** 1 Department of Pharmacology & Toxicology, Brody School of Medicine, East Carolina University, Greenville, NC 27834, USA; robersonj23@students.ecu.edu (J.K.R.); bauera22@students.ecu.edu (A.N.B.); lopezramireza25@ecu.edu (A.L.-R.); jennessd24@students.ecu.edu (D.B.J.); 2 Department of Biology, East Carolina University, Greenville, NC 27834, USA; 3 Department of Biology, University of Puerto Rico at Ponce, Ponce, PR 00716, USA; sebastian.cruz16@upr.edu

**Keywords:** oxidative stress, environmental toxicants, retina, PFAS, microplastics, cigarette smoke, mitochondrial dysfunction, NF-κB, MAPK, ferroptosis

## Abstract

Oxidative stress is a key contributing and convergent pathogenic mechanism linked to retinal and optic nerve diseases including age-related macular degeneration, diabetic retinopathy, and glaucoma. The retina is highly susceptible to redox imbalance due to intense mitochondrial activity, oxygen consumption, and light exposure. While endogenous drivers are well recognized, the contribution of environmental exposure to retinal oxidative injury remains incompletely defined. This review uniquely integrates emerging environmental contaminants with canonical oxidative stress pathways. We examine how cigarette smoke, ultraviolet radiation, heavy metals, microplastics, and per- and polyfluoroalkyl substances (PFASs) promote oxidative injury through mitochondrial dysfunction, inflammatory signaling, impaired antioxidant responses, and ferroptotic pathways. We also highlight therapeutic strategies targeting oxidative pathways and emphasize the importance of exposure-informed retinal and optic nerve disease research.

## 1. Introduction

Retinal and optic nerve diseases, including hypertensive retinopathy (HR), retinitis pigmentosa (RP), age-related macular degeneration (AMD), diabetic retinopathy (DR) [1], and glaucoma [2], are persistent leading causes of visual impairment and blindness in the U.S. [3]. With the growth of the global aging population and increase in metabolic disorders, the burden of retinal pathology continues to rise [4]. Despite advances in therapeutics, mechanisms underlying the molecular processes of damage remain to be elucidated [5,6,7].

A unifying feature across many retinal pathologies is oxidative stress, an imbalance between reactive oxygen/nitrogen species (ROS/RNS) and endogenous antioxidant defenses. The retina is uniquely susceptible to oxidative stress due to its high metabolic rate, dense mitochondrial load, exposure to light, and rich lipid content [8]. When redox homeostasis fails, a cascade of molecular events is triggered, resulting in mitochondrial damage, lipid peroxidation, activation of pro-inflammatory signaling, and ultimately, cell death [8]. Understanding this core axis is essential not just for mechanistic insight. It also represents a convergence point at which environmental exposures can interfere with fundamental cellular defense mechanisms [9,10].

In recent years, environmental risk factors including air pollution [11], ultraviolet (UV) radiation, noise, artificial light, and chemical contaminants have drawn increasing attention for their ability to exacerbate oxidative damage in susceptible tissues. Among these, per- and polyfluoroalkyl substances (PFASs) have emerged as particularly concerning because of their persistence, bioaccumulation, and ability to disrupt redox signaling even at low doses [12,13]. This review narratively explores the continuum from environmental exposure to oxidative stress and subsequent retinal pathology, integrating mechanistic insights, clinical evidence, and preventive strategies. The central pathways linking environmental exposures, oxidative stress, retinal pathology, associated clinical consequences, and potential targeted therapies discussed in this review are summarized schematically in the graphical abstract.

## 2. Oxidative Stress in the Retina: Biological Function

ROS and RNS are continuously generated within retinal tissue as a consequence of normal cellular metabolism and environmental exposure. The primary source of ROS in the retina is mitochondrial oxidative phosphorylation, particularly within photoreceptors, which have one of the highest metabolic demands of any cell type in the body [14,15]. During electron transport, a small but significant fraction of electrons prematurely reduce oxygen to form superoxide (O_2_•^−^), which can subsequently give rise to hydrogen peroxide (H_2_O_2_) and hydroxyl radicals (•OH) [12]. Additional ROS are produced by enzymatic systems such as NADPH oxidases (NOX1-4) and xanthine oxidase [16], while nitric oxide synthases (nNOS, eNOS, iNOS) contribute to RNS formation [17]. Photochemical reactions in the presence of visible and UV light further amplify ROS generation via photosensitization reactions in photoreceptor outer segments [12,18,19].

Under physiological conditions, the retina preserves redox balance through a multilayered antioxidant defense system. Enzymatic antioxidants, including superoxide dismutases (SOD1 in the cytosol and SOD2 in mitochondria), catalase, and members of the glutathione peroxidase (GPx), and peroxiredoxin families, detoxify ROS before they reach damaging concentrations [20]. These enzymes act in concert with non-enzymatic antioxidants such as reduced glutathione (GSH), vitamins C and E, melanin, and the macular carotenoids lutein and zeaxanthin, which mitigate photooxidative stress [21]. Antioxidant responses are further regulated by transcription factors such as nuclear factor erythroid 2–related factor 2 (Nrf2), which upregulates genes involved in GSH synthesis, xenobiotic metabolism, and detoxification [22].

Despite its robust antioxidant defenses, the retina remains particularly susceptible to oxidative stress. Several anatomical and physiological features contribute to this vulnerability. Photoreceptors exhibit exceptionally high oxygen consumption, predisposing them to mitochondrial ROS leakage [14,15]. The outer retina is continuously exposed to light, and the daily phagocytosis of photoreceptor outer segment membranes by retinal pigment epithelial (RPE) cells generates metabolic byproducts capable of redox cycling [23]. In addition, the retina is enriched in polyunsaturated fatty acids (PUFAs) [8], especially docosahexaenoic acid (DHA), which are highly prone to lipid peroxidation [24]. The abundance of iron and other transition metals in the retina further promotes Fenton chemistry, amplifying oxidative injury [25].

Once retinal antioxidant defenses are overwhelmed, redox imbalance triggers a cascade of cellular damage. Lipid peroxidation products, such as 4-hydroxynonenal (4-HNE) [24] and malondialdehyde (MDA) [14], form covalent adducts with proteins, disrupting membrane integrity, and impairing signaling. Protein oxidation leads to enzyme inactivation and proteostasis dysfunction while oxidative DNA lesions, such as 8-hydroxy-2′-deoxyguanosine (8-OHdG), activate repair mechanisms that, when overburdened by extensive damage, may promote cell death [14,26]. Emerging evidence suggests that extracellular vesicles (EVs) can modulate retinal oxidative stress by delivering antioxidant enzymes, regulatory RNAs, and signaling molecules to retinal cells, supporting endogenous defenses and potentially limiting ROS/RNS-mediated damage [27]. Beyond direct biomolecular injury, redox stress also acts as a signaling cue. These signals activate pathways that drive inflammation, metabolic reprogramming, ferroptosis [28], and apoptosis [29]. These downstream events, central to the pathogenesis of many ocular diseases, are examined in the following section.

## 3. Molecular Initiating Events Triggered by Oxidative Stress

Oxidative stress serves not only as a source of molecular damage, but also as a potent activator of cellular signaling. When redox homeostasis is disrupted, retinal cells initiate stress-response pathways that determine whether adaptation, dysfunction, or cell death ensues [30]. These pathways form mechanistic bridges between oxidative imbalance and retinal inflammation, along with retinal diseases such as AMD, DR, HR, RP, and optic nerve diseases such as glaucoma [30]. Understanding these molecular cascades reveals therapeutic targets and clarifies how environmental pollutants accelerate retinal pathology.

### 3.1. Mitochondrial Dysfunction and Metabolic Collapse

Mitochondria are both generators and victims of oxidative stress. Excess ROS impairs electron transport chain complexes, reduces ATP production [31], and induces opening of the mitochondrial permeability transition pore (mPTP) [32]. The resulting release of cytochrome c [33] and other pro-apoptotic factors activates intrinsic apoptosis in retinal ganglion cells (RGCs) and photoreceptors. Mitochondrial dysfunction is a central feature of glaucoma, where disrupted mitochondrial dynamics and defective mitophagy drive progressive RGC loss [34]. Similarly, in AMD, cumulative oxidative damage compromises mitochondrial DNA integrity in RPE cells, leading to bioenergetic collapse and the accumulation of lipofuscin and drusen components [35].

### 3.2. Impaired Antioxidant Defense: Disruption of Nrf2/Keap1 Signaling

Nuclear factor erythroid 2-related factor 2 (Nrf2) is the master regulator of cellular antioxidant defenses. Under oxidative stress, Nrf2 dissociates from its inhibitor Keap1 and translocates to the nucleus, where it induces transcription of antioxidant and detoxification genes, including *HO-1*, *NQO1*, and *GCLC* [22]. In the retina, Nrf2 activation protects against photooxidative damage and hyperglycemia-induced injury. However, during chronic oxidative stress, as seen in diabetic retinopathy, Nrf2 signaling becomes suppressed, leading to glutathione depletion and persistent oxidative injury [36]. Emerging evidence also suggests that environmental toxicants, including PFASs [37] and heavy metals [38], can further inhibit Nrf2 activation, thereby sensitizing retinal cells to redox imbalance.

### 3.3. NF-κB Activation and Inflammatory Amplification

The transcription factor nuclear factor kappa-light-chain-enhancer of activated B cells (NF-κB) is a key mediator of redox-sensitive inflammation. Oxidative stress can activate the IκB kinase complex, allowing NF-κB to translocate to the nucleus and induce expression of pro-inflammatory cytokines (TNF-α, IL-1β) [39], chemokines (MCP-1), and adhesion molecules (ICAM-1) [18,40]. Persistent NF-κB signaling in the retina drives chronic inflammation and microglial activation. In DR, this pathway promotes leukostasis and vascular breakdown, contributing to blood–retinal barrier (BRB) dysfunction [41]. In AMD, oxidative activation of NF-κB enhances complement signaling and inflammasome assembly in RPE cells, linking redox stress to para-inflammation and extracellular deposition [29]. Environmental pollutants such as cigarette smoke extract [42] and diesel exhaust particles [43] also activate NF-κB in retinal cells, underscoring inflammation as a key mediator of pollutant-induced toxicity.

### 3.4. MAPK/JNK/p38 Stress Signaling Pathways

Mitogen-activated protein kinases (MAPKs), including ERK, JNK, and p38 are rapidly activated in response to oxidative stress [44]. JNK and p38 pathways mediate apoptotic signaling and stress-induced autophagy [45]. In models of retinal detachment and ischemia–reperfusion injury, p38 activation promotes photoreceptor apoptosis [46], while JNK contributes to RGC degeneration in glaucoma [47]. The ERK pathway, typically associated with cell survival, becomes maladaptive under sustained oxidative stress, promoting fibrosis and glial scarring in Müller cells [48]. Oxidative activation of MAPK pathways also intersects with NF-κB and ferroptotic lipid signaling, integrating these pathways in a redox-driven injury network [49].

### 3.5. Ferroptosis and Lipid Peroxidation Pathways

Ferroptosis is a regulated form of cell death caused by iron-dependent lipid peroxidation [50]. The retina, rich in polyunsaturated fatty acids and redox-active metals, is especially vulnerable to ferroptotic injury. Glutathione peroxidase 4 (GPX4) is the primary suppressor of ferroptosis by reducing lipid hydroperoxides to alcohol [51]. When glutathione is depleted or GPX4 function is impaired toxic lipid peroxides accumulate, damaging membranes and triggering cell death [52]. Ferroptosis has been implicated in photoreceptor degeneration, light-induced retinal injury [53], and ischemic retinopathy [54]. Notably, features of ferroptosis overlap with AMD pathology, where iron accumulation and lipid peroxidation products (e.g., 4-HNE) are abundant within drusen and RPE deposits [29].

### 3.6. Crosstalk and Outcome Determination

**Figure 1:**
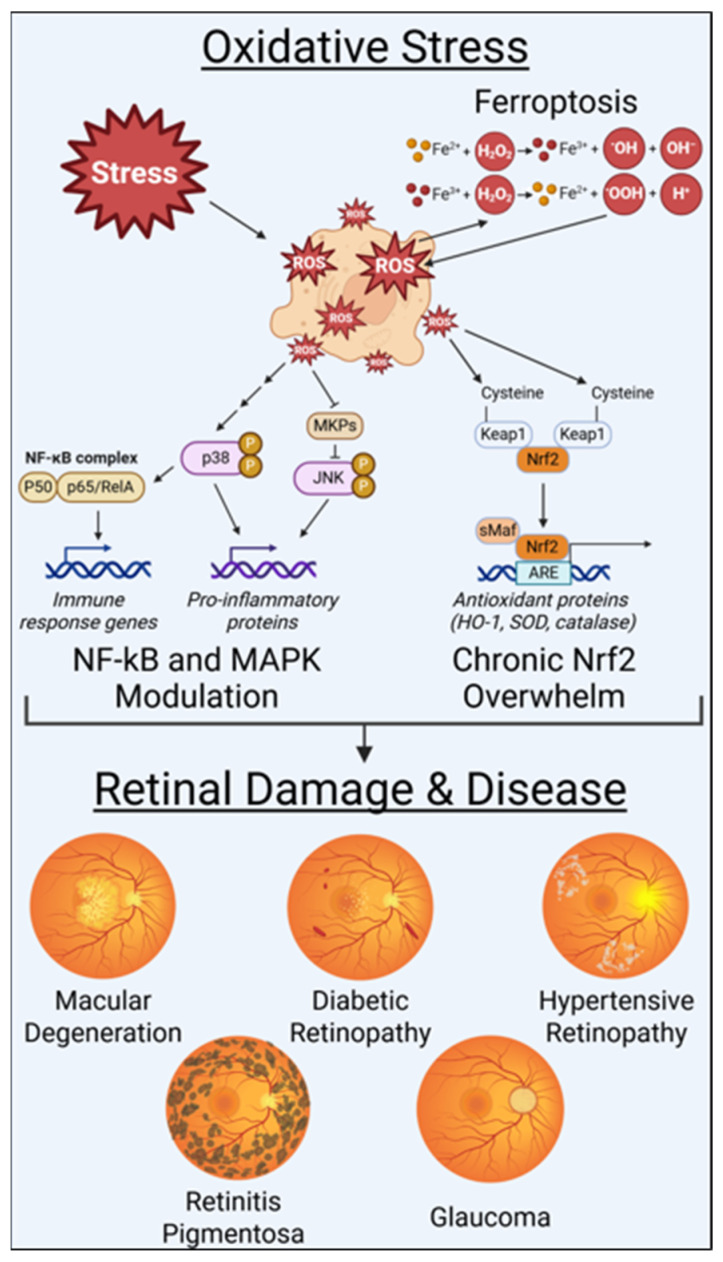
Cellular stress and Fenton reactions increase reactive oxygen species (ROS), which in turn activate molecular pathways involved in inflammation and antioxidant responses. When these protective mechanisms are overwhelmed, oxidative stress can contribute to the development of retinal and other ocular diseases. These pathways represent shared oxidative stress responses across retinal and optic nerve tissues.

Key oxidative stress pathways implicated in retinal and optic nerve pathology, including mitochondrial dysfunction, inflammatory signaling, impaired antioxidant responses, and ferroptosis, are illustrated in Figure 1. These redox-regulated pathways operate within an interconnected network. Mitochondrial dysfunction amplifies ROS production, while NF-κB promotes inflammation and MAPK pathways propagate stress signaling. Ferroptosis further enhances lipid peroxidation, creating a self-reinforcing cycle of damage. The fate of retinal cells, whether survival, adaptation, dysfunction, or death, depends on magnitude and temporal dynamics of these responses. This complex and dynamic molecular interplay provides the foundation upon which aging, genetic predisposition, metabolic disease, and environmental exposures such as PFASs, cigarette smoke, metals, and microplastics converge to accelerate retinal degeneration. These disease-specific manifestations are explored in the following section.

## 4. Clinical Consequences: Oxidative Stress in Retinal and Optic Nerve Disease

Retinal diseases such as AMD, DR, HR, RP, and optic nerve disease such as glaucoma are major causes of vision loss, all sharing oxidative stress as one of many central pathogenic mechanisms. The retina’s high metabolic demand, dense microvasculature, and abundance of polyunsaturated fatty acids render it particularly vulnerable to ROS, which disrupt cellular homeostasis and contribute to diseases such as AMD, DR, and glaucoma [55,56,57].

### 4.1. Age-Related Macular Degeneration (AMD)

In AMD, RPE cellular dysfunction leads to drusen accumulation and photoreceptor degeneration, with advanced disease classified as either neovascular (“wet”) or atrophic (“dry”) AMD. In the wet form, VEGF-driven choroidal neovascularization is exacerbated by ROS and NF-κB–mediated inflammation, while chronic NF-κB activation promotes dry AMD progression and geographic atrophy [56,58]. Anti-VEGF therapy remains the standard of care for wet AMD, though treatment responses vary, and effective interventions for dry AMD are still under investigation [59,60].

### 4.2. Diabetic Retinopathy (DR)

DR arises from chronic hyperglycemia, which promotes excessive ROS generation and damages both retinal neurons and microvasculature. Oxidative stress contributes to microaneurysm formation, vascular leakage, capillary dropout in non-proliferative DR, as well as neovascularization, vitreous hemorrhage, and tractional detachment in proliferative stages [36,61]. Current therapies, anti-VEGF agents, laser photocoagulation, and vitreoretinal surgery, address vascular complications but not the underlying oxidative burden. Transcriptomic profiling is now uncovering redox-related genes as novel therapeutic targets [61,62,63].

### 4.3. Hypertensive Retinopathy (HR)

Although historically considered a vascular disorder, HR is increasingly recognized as a redox-driven disease involving endothelial oxidative injury and mitochondrial dysfunction. Chronic hypertension promotes vascular remodeling and endothelial dysfunction, which are processes closely linked to oxidative stress. Biomarkers such as serum gamma-glutamyl transferase, ferritin, and ischemia-modified albumin increase with disease progression, accompanied by elevated tear fluid glutathione enzymes that may reflect a compensatory antioxidant response [64,65,66]. Further characterization of redox pathways in HR may identify novel therapeutic targets [67].

### 4.4. Retinitis Pigmentosa (RP)

RP is a genetically heterogeneous degenerative disease in which rod photoreceptor death reduces oxygen consumption, creating a hyperoxic environment that amplifies ROS production and accelerate cone loss [68]. Oxidative biomarkers such as 8-oxoG and oxidized glutathione are elevated, while mutations in antioxidant genes (e.g., *MUTYH*, *CERKL*, *GLO1*) compromise retinal resilience [53]. ROS-driven microglial activation further perpetuates inflammation and neurodegeneration [54,69]. Therapeutic approaches that target redox signaling or bolster antioxidant defense may help slow disease progression [70,71].

### 4.5. Glaucoma (Optic Neuropathy)

Glaucoma is a progressive optic neuropathy characterized by RGC degeneration and optic nerve damage to which oxidative stress is a major contributor. Mitochondrial dysfunction, apoptotic signaling, and impaired redox homeostasis drive RGC loss. Elevated oxidative biomarkers, including MDA and protein carbonyls, as well as reduced total antioxidant capacity, correlate with disease severity. Genetic predisposition and microRNA-mediated regulation of redox pathways further heighten vulnerability to ROS [8,72,73]. Both dietary and pharmacological antioxidant strategies have been proposed to slow progression [74,75].

Collectively across these retinal disorders, oxidative stress is a unifying pathogenic driver. Mitochondrial dysfunction, ROS-induced cellular injury, chronic inflammation, and impaired antioxidant defense constitute overlapping mechanisms of degeneration. These shared pathways underscore the therapeutic promise of targeting oxidative pathways across multiple retinal pathologies.

## 5. Environmental Triggers of Retinal Oxidative Stress

**Figure 2:**
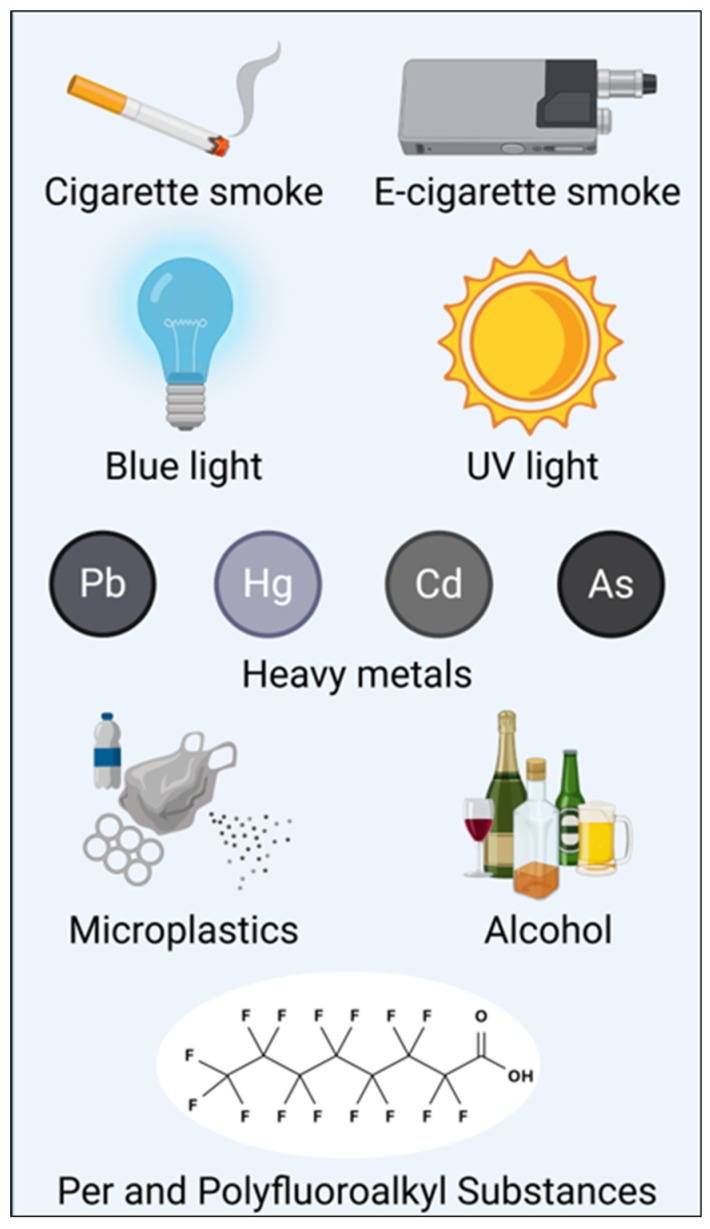
Schematic overview of major environmental and lifestyle exposures associated with oxidative stress in ocular tissues, including the retina and optic nerve. Exposure categories are illustrative and do not imply direct causality or disease specificity.

Environmental exposures are major drivers of ROS generation in retinal tissue and RPE cells. These triggers can be grouped into established toxicants, which are well-characterized contributors to ocular oxidative stress, and emerging contaminants, which are increasingly recognized as potential threats to ocular health. As summarized in Figure 2, diverse environmental and lifestyle exposures contribute to oxidative stress across ocular tissues through overlapping and convergent mechanisms.

### 5.1. Established Toxicants

#### 5.1.1. Cigarette Smoke

Combustible cigarette smoke contains more than 7000 chemicals capable of generating ROS and activating inflammatory signaling [76]. Chronic active or passive exposure induces oxidative damage to DNA, proteins, and lipids, and promotes cytokine-driven inflammation [77,78]. Smoking is a leading modifiable risk factor for AMD; in RPE cells, cigarette smoke triggers mitochondrial damage and ferroptosis through iron accumulation, glutathione depletion, and ROS generation [79]. It is also associated with higher incidence of DR [80] and retinal nerve fiber layer (RNFL) thinning in chronic users [81]. RNFL thinning reflects oxidative and vascular injury to retinal ganglion cell axons, a process that also occurs in glaucoma, and has been observed in patients with Alzheimer’s disease [82], diabetes [83], and hypertension [82], as well as during normal aging [83]. Thus, cigarette smoke-induced oxidative stress represents a convergent mechanism linking environmental exposure, neurodegeneration, and vascular dysfunction within the retina.

#### 5.1.2. Light Stress and Ultraviolet (UV) Radiation

Chronic exposure to UV and high-energy visible (HEV) blue light promotes oxidative stress in ocular tissues. UVA (320–400 nm) and UVB (280–320 nm) radiation stimulate ROS through NADPH oxidase activity, mitochondrial dysfunction, and NF-κB signaling [84]. In the eye, blue light induces mitochondrial membrane depolarization and p38 MAPK activation in RPE cells [85]. UV exposure increases apoptosis-related gene expression [86], linking cumulative light exposure to retinal degeneration.

#### 5.1.3. Heavy Metals

Exposure to cadmium, arsenic, lead, and mercury occurs via contaminated water, food, and air [87]. Redox-active metals (iron, copper) catalyze Fenton chemistry, whereas redox-inactive metals (cadmium, arsenic) deplete glutathione and disrupt antioxidant defense [88]. Heavy metals are linked to retinal toxicity, including thinning of the RNFL and macula [89]. Mechanistically, arsenic impairs the mitochondrial electron transport chain, reduces ATP production, and increases cytochrome c release, triggering apoptosis [90]. Epidemiological data also associate heavy metal burden with elevated risk of DR and poorer glycemic control [91].

### 5.2. Emerging Contaminants: New Environmental Threats

Although established toxicants are well documented, recent environmental shifts have introduced emerging contaminants that may contribute to retinal oxidative stress, yet remain understudied in ocular toxicology [92].

#### 5.2.1. Per- and Polyfluoroalkyl Substances (PFASs)

PFASs are persistent environmental chemicals found in water, air, and consumer products. They bioaccumulate in tissues, including the eye [93], where they disrupt lipid metabolism, mitochondrial function, and redox homeostasis [94]. PFAS exposure increases ROS production in vitro [19] and can activate compensatory oxidative pathways such as Nrf2, depending on dose and exposure duration [95]. The retina is enriched in polyunsaturated fatty acids (PUFAs) [8], especially docosahexaenoic acid (DHA), which are highly prone to lipid peroxidation [23]. Additionally, PFASs downregulate *ABCA1*, a gene essential for RPE lipid efflux and photoreceptor maintenance [96].

#### 5.2.2. Microplastics

Microplastics (<5 mm) are ubiquitous environmental pollutants entering the body through ingestion, inhalation, and dermal contact, having recently been detected in contact lenses and eye care products [97]. Microplastics induce ROS formation and activate p38 MAPK signaling [98], suggesting potential retinal toxicity, although direct evidence in retinal tissue remains limited. Their ability to absorb and transport other pollutants raises additional concern for additive oxidative burden. Although direct evidence in retinal tissue remains limited, convergence on established oxidative and MAPK-mediated stress pathways supports biological plausibility for retinal injury.

#### 5.2.3. Vaping

Electronic cigarettes, marketed as safer alternatives to cigarette smoking [99,100,101], are linked to systemic inflammation, mitochondrial dysfunction, and oxidative stress [102]. Flavorants such as vanillin and cinnamaldehyde induce ROS and impair wound healing in RPE cells [103]. In vivo vape exposure increases pro-angiogenic and pro-inflammatory mediators in the retina [104], mechanisms strongly tied to oxidative disease progression, in experimental models.

#### 5.2.4. Alcohol

Alcohol activates P450 2E1 (CYP2E1), leading to the production of ROS and causing mitochondrial stress in retinal tissues [105]. Ocular MDA levels rise after ethanol exposure [106], and chronic alcohol use reduces mitochondrial respiratory efficiency, promoting oxidative retinal damage [107]. While ocular epidemiological data are limited, mechanistic evidence supports alcohol-induced oxidative stress as a retinal risk factor.

Established and emerging environmental toxicants converge on mitochondrial dysfunction and redox imbalance in retinal tissues, overwhelming endogenous defenses. Traditional exposures (cigarette smoke, UV/blue light, heavy metals) promote ROS overproduction, lipid peroxidation, cytokine activation, and mitochondrial injury. Emerging contaminants (PFASs, microplastics, vaping aerosols, alcohol) show similar capacities to impair mitochondrial bioenergetics, activate oxidative signaling, and disrupt repair processes in RPE cells. Despite clear mechanistic overlap, these newer toxicants remain underrepresented in ocular toxicology research. Defining how diverse exposures converge on redox biology and mitochondrial vulnerability in the retina is essential for risk prediction and prevention.

## 6. Therapeutic Modulation of Oxidative Stress Pathways

Recent advances in molecular therapeutics highlight redox-sensitive pathways that can be targeted to restore cellular resilience and prevent vision loss. Most evidence for these interventions derives from preclinical and experimental models, with limited validation in human retinal and optic nerve disease.

### 6.1. Targeting Mitochondrial Dysfunction

Mitochondrial dysfunction contributes to retinal degeneration via impaired ATP production, excess ROS, and disrupted dynamics. Inhibiting mitochondrial fission (e.g., Drp1 inhibition with mdivi-1) reduces excessive fission and subsequent ROS release [108]. Enhancing fusion is another strategy; metformin promotes AMPK-mediated mitochondrial fusion and protects retinal cells from oxidative injury [109]. Mitochondrial-directed antioxidants also show benefit in preclinical retinal models: gastrodin, derived from herbal tubers, prevents ROS-induced membrane depolarization via 4-HNE suppression [22], and artemisinin reduces mitochondrial apoptosis by modulating ERK1/2/p38 signaling in response to H_2_O_2_ stress [110].

### 6.2. Nuclear Factor Erythroid 2–Related Factor 2 (Nrf2) Activators

Nrf2 orchestrates cellular antioxidant defense. Under oxidative stress, Nrf2 dissociates from Keap1, translocates to the nucleus, and induces antioxidant enzyme expression. Multiple pharmacologic and natural agents enhance Nrf2 signaling in experimental systems by inhibiting Keap1 or stabilizing Nrf2 nuclear translocation [111,112]. However, excessive Nrf2 activation may be detrimental. Overexpression can activate Klf9, a pro-inflammatory transcription factor that elevates ROS and stimulates NF-κB signaling [113,114]. These findings underscore the need for balanced modulation of the Nrf2 pathway.

### 6.3. NF-κB Inhibitors

The transcription factor NF-κB is a central mediator of inflammation and oxidative stress. Inhibition of upstream signals (e.g., blocking IκBα phosphorylation) suppresses NF-κB activation and lowers ROS and cytokine production [115]. Tetramethylpyrazine inhibits IL-1β–mediated NF-κB activation and reduces ROS while concurrently blocking p38 and JNK/ERK1/2 signaling [116]. Crosstalk with JAK2/STAT3 also regulates NF-κB in ocular hypertension via TRPV4 activation [117]; the peptide R9-SOCS3-KIR blocks JAK/STAT signaling, prevents NF-κB nuclear translocation, and reduces cytokines [39]. Similarly, S100A4 peptide suppresses NF-κB through TLR4 suppression, reducing ER stress and inflammatory mediators in retinal ischemia [118].

### 6.4. Mitogen-Activated Protein Kinase (MAPK) Inhibitors

The MAPK pathways drive inflammation and cell stress in oxidative retinal injury. R9-SOCS3-KIR also inhibits p38 MAPK activation and Nrf2 translocation when delivered as an eye drop [39], supporting translational potential. The selective p38 inhibitor SB202190 reduces TGF-β2–mediated phosphorylation and extracellular matrix remodeling [119]. It also promotes GPX4 upregulation, protecting against ferroptosis [120]. Ro3206145 prevents p38 activation in retinal and optic nerve tissues, preserving neuronal transport and RGC survival [121], while PHA666859 reduces ROS, NOS activity, COX-2 expression, and leukocyte adhesion in DR models [122].

### 6.5. Ferroptosis Regulation

Ferroptosis, an iron-dependent form of cell death triggered by lipid peroxidation, contributes to oxidative retinal damage. Therapeutic approaches emphasize iron chelation and restoration of GPX4 activity. NOX2 suppresses GPX4 and promotes ferroptosis [123]; however, slow-release hydrogen sulfide (H_2_S) donors chelate iron, inhibit NOX2, and restore GPX4 levels in models of elevated intraocular pressure [123]. The iron chelator deferoxamine reduces ferroptosis after retinal ischemia–reperfusion by lowering ferritin, preventing hemochromatosis, enhancing transferrin, and activating the System Xc–GSH–GPX4 axis [124].

Summary: Oxidative stress underlies retinal degeneration via mitochondrial dysfunction, inflammatory signaling, and iron-dependent lipid peroxidation. Therapeutic strategies target these pathways at multiple regulatory levels: (i) mitochondrial modulation to restore bioenergetics and limit fragmentation; (ii) Nrf2 tuning to enhance defenses without provoking ROS; (iii) NF-κB inhibition to reduce inflammatory amplification; (iv) ferroptosis suppression to prevent iron-driven cell death. Future therapies will likely require combination approaches that pair redox control with metabolic support.

### 6.6. Prevention and Mitigation Strategies

**Figure 3:**
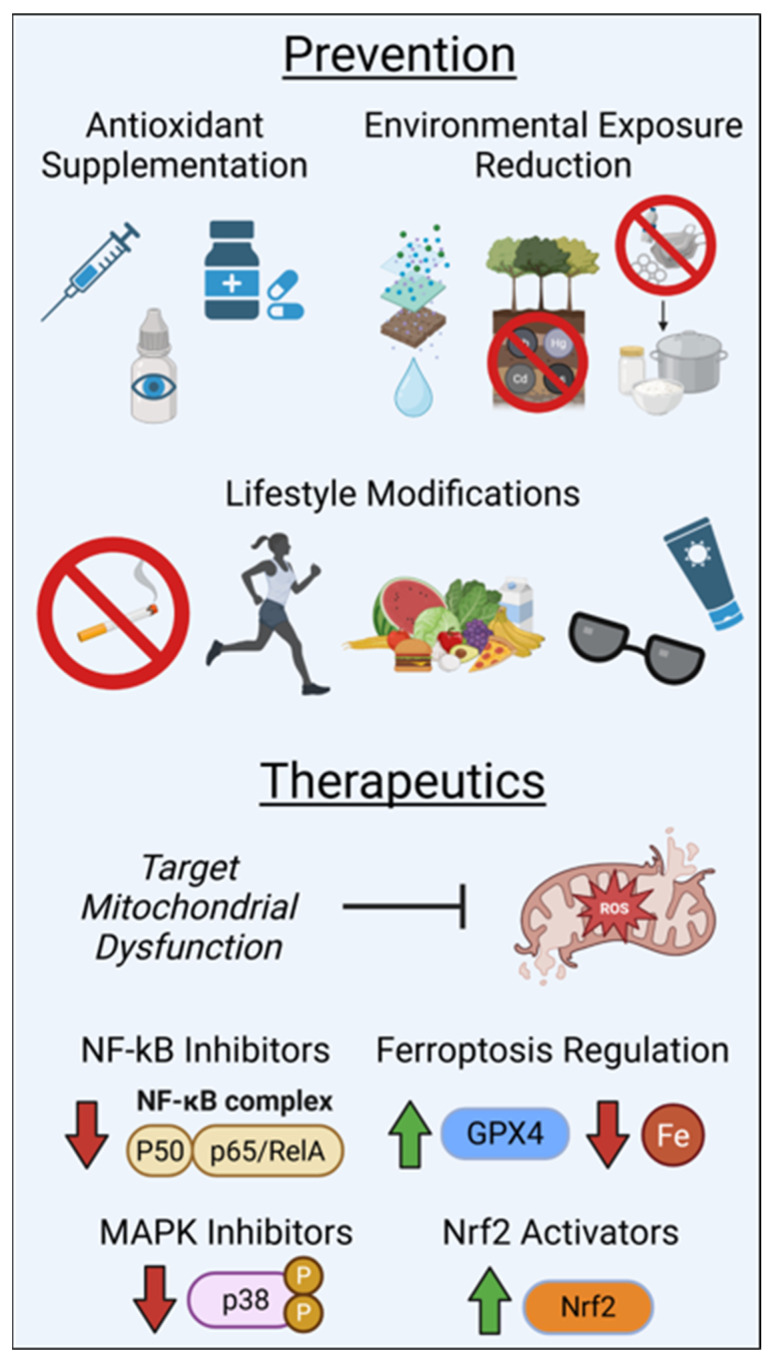
Preventive strategies, lifestyle modifications, and therapeutic approaches targeting oxidative stress pathways in retinal and optic nerve diseases. These interventions are shown as adjunctive strategies that complement disease-specific clinical management.

Both intrinsic and environmental factors contribute to ocular damage, highlighting the need for targeted prevention and mitigation strategies. Preventive strategies and therapeutic approaches targeting oxidative stress pathways in retinal and optic nerve diseases are summarized in Figure 3.

#### 6.6.1. Antioxidant Supplementation

Antioxidant supplementation is best viewed as an adjunctive strategy, rather than a substitute for exposure reduction. Antioxidants counteract ROS/RNS intra- and extracellularly and may mitigate downstream damage in retinal disease models [56]. Experimental strategies include nano-formulated eye drops for glaucoma, intraperitoneal streptozotocin injections to increase glutathione and prevent hyperglycemia in DR, and intravenous cerium oxide (CeO_2_) to reduce microglial activity in oxidative stress-induced AMD [125]. Combined therapies (e.g., coenzyme Q10 plus adjunct antioxidants) reduce systemic lipid peroxidation markers [126], while ferroptosis inhibitors (ferrostatin-1, liproxstatin-1) protect RPE cells by trapping radicals and limiting lipid peroxidation, restoring viability from ~63% to ~102% at 0.5% cigarette smoke extract (CSE), from ~43% to ~100% at 1% CSE, and from ~19% to ~67% at 2% CSE [79].

In addition to conventional antioxidants, several compounds derived from traditional Chinese medicine have demonstrated antioxidant and cytoprotective effects in retinal oxidative injury models. For example, gastrodin [22] and tetramethylpyrazine [116] (see Section 6.1 and Section 6.3), exhibit ROS-scavenging, mitochondrial-stabilizing, and anti-inflammatory properties, supporting their potential role as adjunctive antioxidant strategies in retinal and optic nerve disease.

#### 6.6.2. Environmental Exposure Reduction

Mitigating environmental risk lowers retinal oxidative load. Smoking cessation significantly reduces AMD risk; and physical activity and dietary antioxidants decrease systemic oxidative burden [127]. Protective eyewear against UV (<400 nm) and blue light helps limit light-induced stress [128]. Exposure to heavy metals, microplastics, and PFASs can be curtailed through behavioral and technological measures: soil treatments (e.g., bio-ash amendments) to reduce metal leaching [129], Personal Protective Equipment (PPE) to prevent occupational setting exposure [130], replacing plastic food containers with glass/ceramic/steel [131], using water treatment strategies (e.g., resins, reverse osmosis), and avoiding PFAS-containing products [132,133,134].

#### 6.6.3. Water and Air Remediation Technologies

Emerging technologies aim to reduce environmental toxicant burden. Montmorillonite clay binds with PFASs, decreasing bioavailability and restoring antioxidant levels in experimental models [135,136]. When delivered as eye drops, modified liposomes disperse across the tear film at the water–air interface of the ocular surface, where they reduce ROS levels and inhibit insulin-like growth factor binding protein 6 in retinal tissue [137]. Water treatment technologies, including resin-based filtration and reverse osmosis, efficiently remove PFASs and microplastics, providing an opportunity for population-level exposure reduction.

#### 6.6.4. Lifestyle Strategies to Reduce Oxidative Burden

Dietary patterns rich in antioxidants, regular physical activity, and avoidance of smoking and excessive light exposure remain practical evidence-based approaches to minimize retinal oxidative stress [127].

Prevention strategies that combine antioxidants, lifestyle modification, and exposure reduction can mitigate retinal damage. Emerging technologies in water treatment, remediation, and targeted therapeutics are promising, but further research is needed to refine biomarkers, address complex chemical mixtures, and translate findings to human retinal health.

## 7. Future Directions and Research Gaps

Together, Figure 1, Figure 2 and Figure 3 illustrate how environmental exposures, oxidative stress pathways, and intervention strategies intersect across retinal and optic nerve diseases. Although oxidative stress is a key contributing and convergent pathogenic mechanism of retinal degeneration, important gaps remain in understanding how environmental toxicants, particularly PFASs, interact with redox biology to influence disease progression. Additional mechanistic depth for specific pollutant classes is explored further in a companion review. Most current models rely on high-dose or acute exposures, leaving uncertainty about the cumulative impact of chronic, low-level exposures to PFASs and other emerging contaminants such as microplastics or vaping aerosols. Future research should focus on human-relevant exposure models to better reflect real-world conditions, including chronic, low-dose exposures and physiologically relevant routes of administration. Longitudinal and population-based investigations could help identify better retinal biomarkers of oxidative injury and vulnerability, such as 4-HNE, 8-oxo-dG, or GPX4 activity, while also providing insight into broader patterns of susceptibility or resilience.

Mechanistic gaps remain in linking oxidative signaling networks Nrf2/Keap1, NF-κB/MAPK, and ferroptotic pathways to specific retinal cell types. Emerging technologies, including retinal organoids, advanced imaging, and multi-omics approaches, offer opportunities to map these interactions at high resolution. Cross-tissue comparisons could reveal how systemic exposures affect retinal health relative to other organs, while PFAS and mixture toxicity studies are needed to evaluate the combined effects of multiple environmental stressors. Cross-disciplinary research integrating toxicology, ophthalmology, and environmental health could also explore whether interventions targeting oxidative stress pathways might mitigate injury in broadly applicable ways. Ultimately, combining mechanistic insight with exposure assessment, improved biomarkers, and integrated intervention strategies will be essential for predicting risk, guiding prevention, and informing approaches to PFASs and environmentally mediated ocular diseases.

## 8. Conclusions

Environmental exposures represent an increasingly important and modifiable contributor to oxidative stress across retinal and optic nerve diseases. Both well-established toxicants (e.g., cigarette smoke, ultraviolet and blue light, heavy metals, alcohol) and emerging contaminants (including PFASs, microplastics, and e-cigarette aerosols) converge on shared oxidative mechanisms that disrupt redox homeostasis, impair mitochondrial function, activate inflammatory signaling pathways (including NF-κB and MAPK), dysregulate Nrf2-mediated antioxidant responses, and promote ferroptotic cell death. These processes collectively contribute to neurovascular dysfunction and progressive tissue injury in conditions such as macular degeneration, diabetic and hypertensive retinopathy, glaucoma, and inherited retinal degenerations.

Importantly, oxidative stress should be viewed as a convergent and amplifying mechanism rather than a singular cause of disease. This distinction underscores the need for integrated prevention and mitigation strategies that combine environmental exposure reduction, lifestyle modification, and targeted antioxidant or redox-modulating therapies as adjuncts to disease-specific treatments. Continued efforts to link real-world environmental exposures with mechanistic biomarkers and clinical outcomes will be essential for refining risk assessment, informing public health strategies, and advancing translational approaches aimed at preserving retinal and optic nerve health in an increasingly complex environmental landscape.

## Data Availability

No new data were generated or analyzed in this study. Data sharing is not applicable to this article.
